# Sociodemographic and lifestyle-related risk factors for identifying vulnerable groups for type 2 diabetes: a narrative review with emphasis on data from Europe

**DOI:** 10.1186/s12902-019-0463-3

**Published:** 2020-03-12

**Authors:** Ioannis Kyrou, Constantine Tsigos, Christina Mavrogianni, Greet Cardon, Vicky Van Stappen, Julie Latomme, Jemina Kivelä, Katja Wikström, Kaloyan Tsochev, Anna Nanasi, Csilla Semanova, Rocío Mateo-Gallego, Itziar Lamiquiz-Moneo, George Dafoulas, Patrick Timpel, Peter E. H. Schwarz, Violeta Iotova, Tsvetalina Tankova, Konstantinos Makrilakis, Yannis Manios, on behalf of the Feel4Diabetes-study Group, Yannis Manios, Greet Cardon, Jaana Lindström, Peter Schwarz, Konstantinos Makrilakis, Lieven Annemans, Ignacio Garamendi, Meropi Kontogianni, Odysseas Androutsos, Christina Mavrogianni, Konstantina Tsoutsoulopoulou, Christina Katsarou, Eva Karaglani, Irini Qira, Efstathios Skoufas, Konstantina Maragkopoulou, Antigone Tsiafitsa, Irini Sotiropoulou, Michalis Tsolakos, Effie Argyri, Mary Nikolaou, Eleni-Anna Vampouli, Christina Filippou, Katerina Gatsiou, Efstratios Dimitriadis, Tiina Laatikainen, Katja Wikström, Jemina Kivelä, Päivi Valve, Esko Levälahti, Eeva Virtanen, Ruben Willems, Ivonne Panchyrz, Maxi Holland, Patrick Timpel, Stavros Liatis, George Dafoulas, Christina-Paulina Lambrinou, Angeliki Giannopoulou, Lydia Tsirigoti, Evi Fappa, Costas Anastasiou, Konstantina Zachari, Lala Rabemananjara, Maria Stella de Sabata, Winne Ko, Luis Moreno, Fernando Civeira, Gloria Bueno, Pilar De Miguel-Etayo, Esther Ma Gonzalez-Gil, Maria I. Mesana, Germán Vicente-Rodriguez, Gerardo Rodriguez, Lucia Baila-Rueda, Ana Cenarro, Estíbaliz Jarauta, Rocío Mateo-Gallego, Violeta Iotova, Tsvetalina Tankova, Natalia Usheva, Kaloyan Tsochev, Nevena Chakarova, Sonya Galcheva, Rumyana Dimova, Yana Bocheva, Zhaneta Radkova, Vanya Marinova, Yuliya Bazdarska, Tanya Stefanova, Imre Rurik, Timea Ungvari, Zoltán Jancsó, Anna Nánási, László Kolozsvári, Csilla Semánova, Remberto Martinez, Marcos Tong, Kaisla Joutsenniemi, Katrina Wendel-Mitoraj

**Affiliations:** 1 Aston Medical Research Institute, Aston Medical School, Aston University, B4 7ET, Birmingham, UK; 2 WISDEM, University Hospital Coventry and Warwickshire NHS Trust, Coventry, CV2 2DX UK; 3 Translational & Experimental Medicine, Division of Biomedical Sciences, Warwick Medical School, University of Warwick, Coventry, CV4 7AL UK; 4 Department of Nutrition and Dietetics, School of Health Science and Education Harokopio University, Athens, Greece; 5 Department of Movement and Sports Sciences, Faculty of Medicine and Health Sciences, Ghent University, Ghent, Belgium; 6 Department of Public Health Solutions, National Institute for Health and Welfare, Helsinki, Finland; 7 Department of Paediatrics, Medical University Varna, Varna, Bulgaria; 8 Department of Family and Occupational Medicine, University of Debrecen, Debrecen, Hungary; 9 Unidad Clínica y de Investigación en Lípidos y Arteriosclerosis, Hospital Universitario Miguel Servet, Instituto de Investigación Sanitaria Aragón (IIS Aragón) CIBERCV, Zaragoza, Spain; 10 Universidad de Zaragoza, Zaragoza, Spain; 11 National and Kapodistrian University of Athens, 17 Ag. Thoma St, 11527 Athens, Greece; 12 Department for Prevention and Care of Diabetes, Faculty of Medicine Carl Gustav Carus, Technische Universität Dresden, Fetscherstraße 74, 01307 Dresden, Germany; 13 German Center for Diabetes Research (DZD e.V.), Neuherberg, Germany; 14 Paul Langerhans Institute Dresden of the Helmholtz Center Munich at University Hospital and Faculty of Medicine, Technische Universität Dresden, Dresden, Germany; 15 Department of Diabetology, Clinical Center of Endocrinology, Medical University Sofia, Sofia, Bulgaria

**Keywords:** Type 2 diabetes, Risk factors, Socioeconomic status, Obesity, Lifestyle, Diet, Ethnicity

## Abstract

**Background:**

Type 2 diabetes mellitus (T2DM) comprises the vast majority of all diabetes cases in adults, with alarmingly increasing prevalence over the past few decades worldwide. A particularly heavy healthcare burden of diabetes is noted in Europe, where 8.8% of the population aged 20–79 years is estimated to have diabetes according to the International Diabetes Federation. Multiple risk factors are implicated in the pathogenesis of T2DM with complex underlying interplay and intricate gene-environment interactions. Thus, intense research has been focused on studying the role of T2DM risk factors and on identifying vulnerable groups for T2DM in the general population which can then be targeted for prevention interventions.

**Methods:**

For this narrative review, we conducted a comprehensive search of the existing literature on T2DM risk factors, focusing on studies in adult cohorts from European countries which were published in English after January 2000.

**Results:**

Multiple lifestyle-related and sociodemographic factors were identified as related to high T2DM risk, including age, ethnicity, family history, low socioeconomic status, obesity, metabolic syndrome and each of its components, as well as certain unhealthy lifestyle behaviors. As Europe has an increasingly aging population, multiple migrant and ethnic minority groups and significant socioeconomic diversity both within and across different countries, this review focuses not only on modifiable T2DM risk factors, but also on the impact of pertinent demographic and socioeconomic factors.

**Conclusion:**

In addition to other T2DM risk factors, low socioeconomic status can significantly increase the risk for prediabetes and T2DM, but is often overlooked. In multinational and multicultural regions such as Europe, a holistic approach, which will take into account both traditional and socioeconomic/socioecological factors, is becoming increasingly crucial in order to implement multidimensional public health programs and integrated community-based interventions for effective T2DM prevention.

## Background

Type 2 diabetes mellitus (T2DM) prevalence has been increasing rapidly over the past few decades worldwide [1, 2]. Based on recent International Diabetes Federation (IDF) estimates, in 2017 there were 451 million adults (age: 18–99 years) with diabetes globally, a figure which is predicted to reach 693 million by 2045 [3]. In the region of Europe alone, 58 million adults are estimated to have diabetes and this number is also expected to significantly increase to 66.7 million by 2045 (8.8 and 10.2% of the population aged 20–79 years, respectively), highlighting the heavy healthcare burden of diabetes in Europe [4]. Moreover, it is estimated that almost half of all people with diabetes are undiagnosed (22 million undiagnosed cases in Europe) and, hence, remain untreated and at significantly greater risk of T2DM complications [1, 3, 4].

T2DM results from progressive loss of insulin secretion, which is typically combined with various degrees of insulin resistance [5–7]. An array of risk factors is implicated in the pathogenesis of T2DM, including age, ethnicity, family history, low socioeconomic status, obesity, metabolic syndrome, and certain unhealthy lifestyle behaviors [Table 1] [1, 7–9]. The exact interplay between these T2DM risk factors represents a complex pathophysiologic process with intricate underlying gene-environment interactions, which appear to vary within different populations [1, 8–10]. As such, research interest has been focused towards studying the pathogenetic role of various T2DM risk factors and on identifying vulnerable groups for T2DM which can then be targeted in clinical practice, prevention interventions and public health programs [1, 8–13].

**Table 1:** List of key risk factors implicated in the pathogenesis of type 2 diabetes mellitus (T2DM)

Key risk factors implicated in the pathogenesis of type 2 diabetes mellitus (T2DM)	
▪ Increased body weight - overweight or obesity (body mass index, BMI, ≥25 kg/m^2^ for Caucasian adults) ▪ Increased abdominal/visceral adiposity - central/android obesity, increased waist circumference independent of BMI ▪ Other metabolic syndrome components (e.g. hypertriglyceridemia, low HDL-cholesterol plasma levels, hypertension) ▪ Unhealthy eating/dietary habits (e.g. high consumption of processed red meat, sugar-sweetened beverages and alcohol, and/or low consumption of fruits, vegetables, high-fiber and whole grain foods) ▪ Sedentary lifestyle - decreased physical activity ▪ Cigarette/tobacco smoking ▪ Aging (older age) ▪ Race/Ethnicity (non-white ethnic background/ancestry) ▪ T2DM family history (particularly with first degree relatives and with earlier age of onset) ▪ Genetic predisposition/factors ▪ Gestational diabetes mellitus history ▪ Low socioeconomic status, deprivation ▪ Stress, anxiety and depression ▪ Certain medications (e.g. certain statins and beta-blockers)	

In this article, we present a narrative review of key risk factors and lifestyle behaviors related to high T2DM risk, with reference to relevant findings from studies in adult cohorts from European countries. As Europe has an increasingly aging population, multiple migrant and ethnic minority groups and significant socioeconomic diversity both within and across different countries [3, 4, 14], this review is intended to focus not only on modifiable T2DM risk factors, but also on the impact of pertinent demographic and socioeconomic factors. For example, despite being often underestimated, low socioeconomic status can significantly increase the risk for prediabetes and T2DM, particularly in high-income countries [15], thus, becoming increasingly important for assessing more precisely the risk of T2DM among various European populations. As such, this narrative review provides a comprehensive overview of key sociodemographic and lifestyle-related risk factors for identifying vulnerable groups for T2DM with emphasis on data from Europe.

## Methods

Although this paper is not a systematic review, in the present narrative review we conducted a comprehensive search of the relevant literature published in English after January 2000. To this aim, PubMed was searched using terms relating to T2DM [e.g. diabetes, type 2 (II) diabetes mellitus (T2DM, DM), non-insulin-dependent diabetes mellitus (NIDD, NIDDM), and adult-onset diabetes], T2DM risk factors [e.g. age, BMI, central/visceral/android obesity, metabolic syndrome, family history, race/ethnicity, socioeconomic status/position/indices, SES, low−/middle−/upper-income, poverty, poor, vulnerable, deprivation, education level and risk factor], and diabetes-related lifestyle behaviors [e.g. unhealthy diet, snacking, sweet/soft/fizzy drinks, sugar-sweetened beverages, whole grains, savory/sweet/salty snacks, confectionary, fast food, meal/portion size, exercise, physical activity/inactivity, leisure activity, sedentary/sitting behavior/time, domestic activities, walking, outdoor play, computer use/time, gaming, screen time, reading, and television viewing].

Based on the aforementioned search terms, more than 2250 publications were detected in PubMed, which were then screened by titles and abstracts. Accordingly, pertinent studies with adult cohorts from European countries, and relevant review articles on the etiology of high T2DM risk were identified and obtained. The references of these papers were also hand-searched. In total, more than 140 publications were identified as relevant to the scope of this work, and were reviewed in detail. Of those, 101 publications were included and cited in the present narrative review, highlighting key lifestyle-related and sociodemographic risk factors related to increased T2DM risk in adults with focus on the existing evidence from Europe.

## Results

### T2DM risk factors related to obesity and other metabolic syndrome components

“*Diabesity*” has been introduced as a new term to better describe the twin epidemic of T2DM and obesity [16], since the rising prevalence rates of T2DM during the past decades have been closely tracking those of obesity worldwide [1–4, 17]. Obesity is now recognized as the most important modifiable risk factor for prediabetes and T2DM, which, depending on the degree, distribution, timing and duration of excess weight gain, can progressively lead to a spectrum of metabolic syndrome manifestations and cardiovascular disease [18]. Of note, beyond the impact of increasing body mass index (BMI) on the risk of T2DM, an independent positive association has also been clearly documented between central/visceral obesity and T2DM [16, 18, 19]. As such, visceral and ectopic (e.g. in the liver, skeletal muscles, and heart) fat accumulation are now regarded as key contributing factors to increased T2DM risk, correlating directly with hyperinsulinemia and insulin resistance [18]. A combination of underlying mechanisms appears to explain the higher risk of T2DM with central obesity and ectopic fat accumulation, primarily relating to dysfunction and a markedly altered secretion profile of adipocytes in these fat depots [18]. Indeed, adipocytes in visceral fat depots are more lipolytic, whilst they also exhibit enhanced secretion of pro-inflammatory adipokines (e.g. intrerleukin 6 and tumor necrosis factor-α) and decreased secretion of anti-inflammatory adipokines (e.g. adiponectin), thus, promoting increased insulin resistance and activation of inflammatory pathways in the adipose tissue, liver and skeletal muscles [18]. These characteristics of visceral and ectopic fat deposition contribute to the development of the cardio-metabolic complications of obesity (particularly of central obesity), as has been consistently documented in both basic and clinical research studies [18].

#### Obesity - body mass index (BMI)

The relative risk for T2DM in adults rises markedly with increasing BMI over 30 kg/m^2^, while it appears to begin to increase even within the normal BMI range (from 22 and 24 kg/m^2^ for women and men, respectively) [19, 20]. Multiple studies in Europe have examined the association between objectively measured BMI and T2DM, showing that increased BMI holds a significant positive association with T2DM [21–25]. In this context, Wannamethee et al. have further reported significantly increased T2DM risk with both overweight and obesity during the 20-year follow-up of the prospective British regional heart study (7735 men; age: 40–59 years) [23]. Furthermore, BMI has been reported to independently predict the development of T2DM, with incidence rates that were approximately three and ten times higher in individuals with BMI ranging from 25 to 30 kg/m^2^ and over 30 kg/m^2^, respectively [24]. However, it should be noted that other data show that BMI is not an independent T2DM predictor [25], indicating that, when variables such as waist circumference and waist-to-hip ratio (WHR) are taken into account, central obesity is a better T2DM predictor [22, 25].

#### Central obesity - abdominal/visceral fat distribution

A consistent positive association has been documented between central/visceral obesity and T2DM risk, independent of BMI [18, 26, 27]. As aforementioned, this strong correlation between visceral fat accumulation and both insulin resistance and hyperinsulinemia is primarily attributed to a more deleterious secretory, lipolytic and pro-inflammatory profile of adipocytes in visceral fat depots [18]. As increased visceral adiposity is now recognized as an independent T2DM risk factor [18], certain anthropometric indices of central obesity (e.g. waist circumference, WHR and waist-to-height ratio) are now applied in clinical practice and research to better assess the obesity-related risk of T2DM [26–29]. Waist circumference represents the simplest and most extensively studied among these anthropometric indices, with numerous studies on the relationship between objectively measured waist circumference and T2DM risk in various European populations [21, 22, 25–31]. These studies showed that the prevalence of T2DM rises with increasing waist circumference, and that the latter is a significant independent predictor of T2DM development even after adjusting for BMI [21, 22, 25–31].

#### Weight gain

Sustained weight gain during various periods of the adult life appears to further impact on the T2DM risk, with the results from the European Prospective Investigation into Cancer and Nutrition (EPIC)-Potsdam Study showing that weight gain in early adulthood (age: 25–40 years) is associated to higher T2DM risk and earlier T2DM onset compared to weight gain later in life (age: 40–55 years) [32]. Substantial weight gain (> 10%) during the long-term follow-up of the prospective British regional heart study was also related to increased T2DM risk [23]. Moreover, the prospective Doetinchem Cohort Study (1987–2007) showed that a 5-year weight change in adults (age: 20–59 years) was associated with the T2DM incidence in the subsequent 5 years, even after adjustment for the initial BMI [33]. However, in this Dutch population-based study there was no significant association between weight change and T2DM after adjusting for the attained BMI, suggesting that such weight changes may not exert an independent impact on the T2DM incidence beyond their effect on the attained BMI [33].

#### Other metabolic syndrome components

The association between T2DM risk and additional metabolic syndrome components (e.g. hypertension and dyslipidemia) has been also investigated in various European studies [22, 24, 34]. As such, data from studies in Italy and Greece showed 2- to 3-fold higher T2DM incidence in patients with hypertension (systolic blood pressure ≥ 140 mmHg or diastolic blood pressure ≥ 90 mmHg or treatment for hypertension) compared to normotensive individuals, further indicating that hypertension is independently associated with higher T2DM risk [22, 24]. Similarly, these studies also found a positive association between dyslipidemia and T2DM [22, 24]. Indeed, Melidonis et al. reported that fasting triglyceride plasma levels were independently associated with 3.6-fold increased T2DM risk [22], whilst Bonora et al. showed that the T2DM incidence was approximately 1.5-fold higher in individuals with dyslipidemia [defined as fasting triglycerides ≥1.7 mmol/l (150 mg/dl) and/or HDL-cholesterol ≤1 mmol/l (40 mg/dl) in women or ≤ 0.9 mmol/l (35 mg/dl) in men] compared to those with normal triglycerides and HDL-cholesterol levels [24].

Overall, it is worth highlighting that the metabolic syndrome represents a constellation of independent cardio-metabolic risk factors, including obesity (particularly with central/visceral fat deposition), hypertension, dyslipidemia, and glucose intolerance, which cluster together and correlate with higher T2DM and CVD morbidity and mortality [18]. Indeed, the presence of the metabolic syndrome appears to result in a 1.5-fold increase in all-cause mortality and a 2-fold increase in CVD outcomes, whilst it also linked to additional complications, such as obstructive sleep apnea (OSA), non-alcoholic fatty liver disease (NAFLD) and non-alcoholic steatohepatitis (NASH), which further contribute to higher T2DM and CVD risk [18]. Notably, based on meta-analysis data from 16 cohorts with 42,419 participants, an average estimated relative risk of 3.5–5.2 was documented for the association of metabolic syndrome with incident diabetes, indicating that the metabolic syndrome constitutes a key predictor of incident diabetes in various populations, including Europeans and those of European descent [35].

### T2DM risk factors/behaviors related to diet and physical activity.

Unhealthy dietary habits, sedentary lifestyle and decreased physical activity are closely associated with increased T2DM risk both directly and indirectly through promoting obesity and various metabolic syndrome components [Table 1] [1, 8, 9]. As such, lifestyle modification that aims to improve dietary habits and increase physical activity levels constitutes the cornerstone of T2DM prevention interventions [8]. Indeed, the Finnish diabetes prevention program resulted in 58% reduced T2DM risk in overweight adults (40–65 years old) with impaired glucose tolerance that received individualized counseling aiming to reduce body weight and both total and saturated fat intake, as well as to increase fiber intake and physical activity [36]. Notably, such interventions have been shown to have sustained long-term benefits (e.g. healthier dietary habits, and lower body weight, fasting and postprandial glucose levels) for many years following the active intervention period [8, 37].

#### Diet - dietary patterns

Overall, high consumption of sugar-sweetened beverages, processed red meat, refined grains and alcohol, as well as diets low in fruits, vegetables, fiber and wholegrain foods are linked to higher T2DM risk [1, 8, 9, 38]. Notably, such associations (e.g. the association between higher intake of sugar-sweetened beverages and increased T2DM risk) appear to remain significant even after controlling for BMI [38]. Furthermore, improving the quality of the carbohydrates and fats in the diet (e.g. following diets low in glycemic load, glycemic index and trans fatty acids, and high in cereal fiber, polyunsaturated fatty acids and polyunsaturated-to-saturated fat ratio) appears to favor T2DM prevention, suggesting that the quality of these macronutrients is also a significant T2DM risk factor [38]. In addition to the quantity and quality of macronutrients in the diet, recent interest has also focused on certain dietary patterns (e.g. the Mediterranean diet) which can lower the T2DM risk as part of a comprehensive dietary approach rather than concentrating on single dietary components [39, 40]. The Mediterranean diet constitutes the traditional dietary pattern followed during the middle of the previous century in olive-growing areas of the Mediterranean region (e.g. in southern Italy, Spain and Greece), and is characterized by olive oil consumption as the main fat source, high consumption of plant-based foods and wholegrain cereals and bread, low-to-moderate consumption of fish, poultry, dairy products and wine with meals, as well as low consumption of processed and red meat [40]. A growing body of evidence from both prospective studies and randomized controlled trials has shown that higher adherence to the Mediterranean diet is linked to significantly reduced T2DM risk, even after adjustment for potential confounders (e.g. BMI) [40–44]. Moreover, systematic review data from prospective studies have shown that adherence to the Dietary Approaches to Stop Hypertension (DASH) and the Alternative Healthy Eating Index (AHEI) may also lower the risk of T2DM [39], suggesting that such dietary patterns can be also effective for T2DM prevention [39, 40]. Interestingly, analyses of data from seven European countries of the EPIC-InterAct case-cohort study revealed no significant association between the DASH or AHEI and T2DM risk after multivariable adjustment which accounted for body size [45]. However, in this study lower T2DM risk was noted with adherence to certain reduced rank regression (RRR)-derived dietary patterns characterized by low intake of sugar-sweetened beverages, processed meat, and refined grains, as well as high intake of vegetables or fruits [45]. The stronger association of these RRR-derived dietary patterns to T2DM risk compared to the DASH and AHEI can be potentially attributed to the fact that the former were specifically derived to explain variations in T2DM-relevant biomarkers [45]. In this large European study with both Mediterranean and non-Mediterranean countries, adherence to the Mediterranean diet, as assessed by the relative Mediterranean diet score, was also associated with a small reduction in the risk of T2DM [46]. Notably, this association was attenuated in adults with age less than 50 years or obesity, as well as when the olive oil, meat and alcohol components were excluded from the assessed score [46]. Findings such as those from the EPIC-InterAct study further highlight the need to take into account the potential interplay between multiple factors/behaviors which can impact on the risk of developing T2DM.

#### Physical activity - sedentary behaviors/lifestyle

Increased sedentary time and decreased physical activity levels contribute to high risk of prediabetes and T2DM [1, 8, 9, 47]. In 2016, based on a pooled analysis of 358 population-based surveys with 1.9 million participants across 168 countries, the global age-standardized prevalence of insufficient physical activity was 27.5%, with even higher prevalence in women and high-income countries [48]. Recent data based on objectively measured physical activity and sedentary behaviors showed that the vast majority (> 95%) of the adults in the EPIC-Norfolk Study (1259 participants; age: 49–91 years) were insufficiently active, with increasing sedentary time and decreasing total, light- and moderate-to-vigorous physical activity over time [49]. In this study, correlates of higher rates of the increase in sedentary time and the decrease in physical activity included both higher BMI and older age in men and women [49]. Combating such alarming trends is an important component of T2DM prevention, particularly in individuals at high T2DM risk [47]. Overall, a recent Cochrane systematic review of randomized controlled trials with duration of at least two years showed that combining diet with increased physical activity delays the development and reduces the risk of T2DM in adults with impaired glucose tolerance [50]. However, further evidence is still needed regarding the impact of physical activity alone [50]. Furthermore, data from the ProActive UK trial in adults with T2DM family history showed that objectively measured moderate- and vigorous-intensity physical activity predicted insulin resistance independent of sex, age, waist circumference, smoking status, self-reported TV viewing and time spent sedentary and at light-intensity activity; thus, highlighting the benefits of promoting at least moderate-intensity physical activity (e.g. brisk walking) on insulin sensitivity in such high-risk populations [51]. As such, structured lifestyle interventions which include at least 150 min of regular moderate-to-vigorous physical activity per week and dietary changes aiming in 5–7% weight loss are recommended in order to delay or prevent T2DM in adults at high T2DM risk [47]. Moreover, a population-based study in middle-aged adults with 5.6 years of follow-up (Medical Research Council Ely study, 1994–2003) showed that objectively measured sedentary time may also predict insulin resistance at follow-up, even after adjustment for moderate- and vigorous-intensity physical activity levels, and independently of baseline age, sex, fat mass, smoking status, fasting insulin levels and follow-up time [52]. Thus, reducing sedentary behaviors, which are characterized by energy expenditure of ≤1.5 metabolic-equivalent units and are conducted in sitting, reclined or lying down positions (e.g. television viewing and desk work), is also recommended as an additional measure on top of structured exercise in order to further decrease the risk of developing T2DM [47].

### T2DM risk factors related to socio-demographic parameters

Taking into account non-modifiable T2DM risk factors (e.g. age, family history and ethnicity), as well as socioeconomic-related parameters, is an integral part of the risk assessment process to effectively identify individuals and sub-group populations at high T2DM risk which should be targeted for intensive interventions and prevention programs [1, 9, 53].

#### Aging - older age

Life expectancy has been rising in most countries, with the global life expectancy at birth having increased from 65.6 years in 1990 to 73 years in 2017 [54]. After superimposing these trends for progressively aging populations worldwide to the global diabesity epidemic and the recent advances in the survival of patients with diabetes, it is no surprise that older age is now recognized as an increasingly crucial T2DM risk factor [2–4, 54, 55]. Aging increases the risk of developing T2DM by both impairing insulin secretion and enhancing insulin resistance through obesity and sarcopenia [55]. In addition, increasing age has also been shown to independently predict lower daily physical activity levels [56], with the rates of insufficient physical activity, increasing sedentary time and decreasing physical activity being more pronounced among older adults [47]. Currently, older adults (age: ≥65 years) exhibit the highest T2DM prevalence among any age-group, whilst the diabetes cases in this age-group worldwide are expected to increase from 122.8 million in 2017 to 253.4 million in 2045 [3]. Based on the diabetes prevalence data by World Bank income group and age, middle- and high-income countries exhibit the highest diabetes prevalence in the 60–74 (19%) and 75–79 (22%) age-group, respectively, while in low-income countries the peak diabetes prevalence (8%) is documented in the 55–64 age-group [3]. Of note, high-income countries appear to have 3-fold higher diabetes prevalence among the 65–69 age group compared to low-income countries [3]. Particularly in Europe, where the general population aged 50–99 years is projected to reach 53.6% by 2045 (45.1% in 2017), aging is considered a key driver of the T2DM epidemic which accounts for a large proportion of the current (19.4%, 28.5 million cases in 2017) and projected (19.8%; 43.9 million cases in 2045) high diabetes prevalence among people older than 65 years [4]. This association between aging and T2DM has been confirmed in both men and women by multiple European cross-sectional and longitudinal studies, which have consistently showed that age is an important T2DM predictor with significantly higher T2DM prevalence with increasing age [4, 22, 24, 25, 30]. Finally, although Europe exhibits one of the lowest proportion of undiagnosed diabetes cases (37.8%) worldwide, a large number of older adults with diabetes remains undiagnosed [3, 4], whilst many older adults with diabetes who receive care at home are also unaware of their diagnosis [57].

#### Diabetes family history

Family history of T2DM is recognized as a crucial non-modifiable T2DM risk factor, constituting an easily assessed marker of the underlying T2DM genetic predisposition [1, 58, 59]. Indeed, multiple studies with European cohorts reported that T2DM family history is an independent predictor of T2DM in both men and women [22, 25, 59–63]. Notably, data from six European countries of the EPIC-InterAct study showed that T2DM family history was strongly associated with the T2DM risk, with most of this risk remaining unexplained even after accounting for key anthropometric, lifestyle and genetic T2DM risk factors (e.g. BMI, waist circumference, physical activity and a multi-SNP genetic risk score) [59]. Furthermore, analyses from the Dutch contribution to the EPIC study (EPIC-NL; 35,174 adults; age: 20–70 years; median follow-up:10.2 years) documented a positive association between both paternal and maternal diabetes history and increased T2DM risk, independent of lifestyle, diet and adiposity [60]. Based on these data, history of maternal diabetes conferred slightly higher T2DM risk compared to paternal, due to larger contribution of lifestyle factors, diet and adiposity [60]. Interestingly, in two population-based cross-sectional surveys on T2DM in Finland and China (FINRISK 2002 Study and the Qingdao Diabetes Prevention Program, respectively) which are included in the DECODE-DECODA collaboration (Diabetes Epidemiology: Collaborative Analysis of Diagnostic Criteria in Europe and Asia), the synergistic effect of diabetes family history with obesity on the prevalence of T2DM was significant only in Finnish men and not in the Chinese cohort [61]. Moreover, findings from a large cohort of 60-year old men and women from Sweden suggest that history of parental diabetes in combination with obesity appears to be particularly hazardous in men by synergistically increasing the risk of developing T2DM [62]. It is also noteworthy that, the Botnia Study in Finland (5810 individuals from 942 families) reported that the strongest T2DM heritability was identified in patients with onset age between 35 and 60 years [64]. Overall, there is now compelling evidence from studies with family and twin cohorts that genetic factors strongly impact on the T2DM risk, whilst a rapidly increasing body of large genome-wide association studies (GWAS) has identified over 300 genetic variants which are strongly associated with T2DM [1, 65]. However, due to the highly complex and polygenic T2DM nature, these explain only a fraction of the T2DM heritability, while the rest could be attributed to other factors (e.g. gene-environment interactions between T2DM-related genetic loci and environmental exposures/determinants) [1, 5]. In fact, recent analysis within the EPIC-InterAct study across eight European countries has shown that the genetic susceptibility to T2DM, insulin resistance and BMI did not modify the association between macronutrient intake and incident T2DM, suggesting that the macronutrient intake recommendations for T2DM prevention do not require further adjustment for differences in this genetic predisposition [66]. Similarly, recent data from the Malmö Diet and Cancer cohort showed that both a genetic-based and a diet-based T2DM risk score were associated with increased T2DM incidence, but apparently in an independent way, thus supporting the notion that individuals at either high or low genetic risk would benefit from favorable diet choices [67].

*Ethnicity*: Ethnicity constitutes an additional non-modifiable T2DM risk factor, with certain ethnic groups exhibiting inherently higher T2DM risk regardless of the country of residence [1, 9, 18, 68]. Apart from differences in lifestyle and socioeconomic related parameters, these ethnic-related disparities in the T2DM prevalence are attributed to higher T2DM genetic predisposition and enhanced susceptibility for cardio-metabolic complications in relationship to body composition, central fat distribution and obesity [1, 18, 68–70]. As such, significantly higher risk for insulin resistance and T2DM has been noted in South Asian, Chinese and Japanese individuals with overweight or obesity compared to weight-matched controls of Caucasian origin [68–73]. In Europe, several studies have investigated the prevalence of T2DM in migrant and ethnic minority groups compared to European host populations, with the accumulated evidence consistently showing higher T2DM prevalence among the former groups [68, 72–79]. For example, in the UK the T2DM prevalence among minority ethnic communities is approximately 3- to 5-fold higher than in the white British population, with earlier onset by 10–12 years and a significant proportion of T2DM patients aged less than 40 years [73]. Overall, systematic review and meta-analysis data have shown that the T2DM risk among ethnic minority groups living in Europe is higher compared to European host populations and varies according to their geographical origin [68]. Specifically, the groups of South Asian, Sub-Saharan African, and Middle Eastern/North African origin exhibit, respectively, 3- to 5-fold, 2- to 3-fold, and 2- to 4-fold higher T2DM risk (higher for women than for men) [68]. Furthermore, among the South Asian sub-groups, Bangladeshi had the highest T2DM risk compared to Europeans, followed by Pakistani and then Indians [68]. Based on nationwide data on the asylum seekers who arrived in the Netherlands from 2000 to 2008, higher T2DM risk has been reported in asylum seekers (aged 20–79 years) from most of the origin countries (particularly from Somalia, Sudan and Sri Lanka) compared to the general Dutch population [79]. Such data are becoming increasingly pertinent, since a growing burden of diabetes in migrant minority populations is now posing a public health challenge in several European countries, while the migration flow of people into Europe is not expected to slow down soon as it is driven by both “pull-factors” in host countries (e.g. job opportunities and better well-being) and “push-factors” in native/origin countries (e.g. food shortage, economic crisis, terrorism and wars) [14]. In this context, it becomes evident that policy initiatives and public health programs for T2DM prevention should further take into account the ethnic-related differences in T2DM risk among the increasingly diverse populations within and across different countries.

#### Socioeconomic status

Low socioeconomic status (SES), assessed mainly based on income, occupation and educational level, is an independent T2DM risk factor [Table 1] [9, 15, 80–84]. Systematic review and meta-analysis data have shown that increased T2DM incidence is associated with low SES in high-, middle- and low-income countries [15]. Notably, the strength of this association was consistent in high-income countries, but further evidence is needed for low- and middle-income countries [15], since for the latter there are also data suggesting higher T2DM prevalence in high SES groups [80]. The exact mechanisms linking low SES to increased T2DM risk are still under investigation, with key modifiable T2DM factors (e.g. obesity, diet, physical activity and alcohol intake) accounting for 33 to 50% of this association [83]. The remaining part may be attributed to various other factors relating to psychosocial stress, hopelessness, material deprivation, restricted autonomy, and limited access to healthy food, exercise facilities and health services [83–85].

In Europe, the significance and magnitude of the association between deprived SES and T2DM vary depending on the study, with part of these inequalities explained by SES differences in the prevalence of traditional T2DM risk factors [85]. A large French survey (32,435 men and 16,378 women, age: 35–80 years) investigated the relationship between T2DM and SES, as assessed by the EPICES score which accounts for the multidimensional material, psychological and social aspects of deprivation [86]. The findings of this study showed higher risk of T2DM onset among deprived men and women compared to non-deprived subjects, even after accounting for age, and lifestyle, clinical and biological variables [86]. Another cross-sectional and longitudinal study in 11 European countries in 2004 and 2006 (21,323 men and women, aged ≥50 years) revealed that only women had significant inequalities in the T2DM prevalence and incidence as a function of SES, which were mediated by BMI [87]. Indeed, the available evidence in Europe suggests that the SES inequalities in T2DM tend to be greater and more consistent in women compared to men [85, 88]. Moreover, a cross-sectional study in middle-aged Swedish adults (3128 men and 4821 women, age: 35–56 years) showed that the association between T2DM and low SES early in life (fathers’ occupation and participants’ education) disappeared after adjusting for the adult SES (participants’ occupation) and adult T2DM risk factors [89]. However, systematic review data indicate that childhood SES is associated with T2DM in later life, since this association remains despite the fact that adjustment for adult SES and obesity tends to attenuate the T2DM risk attributed to childhood SES [90].

It should be also highlighted that, cross-sectional data from five German population-based studies (11,688 men and women, age: 45–74 years) showed a stepwise increase in T2DM risk with increasing regional area deprivation, independent of individual SES [91]. Interestingly, the long-term follow-up of the “Moving to Opportunity” project, which was a social experiment designed and implemented by the US Department of Housing and Urban Development, revealed that moving from a high- to a lower-poverty neighborhood was associated with modest, but potentially important decrease in the prevalence of both diabetes and extreme obesity [92]. Furthermore, findings from the 2002 Oslo Health Study suggest that the residential location (organization of urban space and spatial distribution of health-related resources) of individuals is a highly significant and independent T2DM predictor even after adjusting for multiple covariates (e.g. age, BMI and ethnicity) [93]. Thus, living in the east side of Oslo, which is a disadvantaged and densely populated area associated with social stigma and immigration, was found to increase the odds of T2DM by approximately 60% [93]. In this study, the positive association between T2DM and ethnicity was, at least, partly mediated by the local sociospatial/environmental setting [93]. Another study from Sweden also reported high T2DM prevalence in a socially vulnerable neighborhood, independent of the country of origin (Sweden or Iraq) [94]. Similar data have been also reported in the US, with the community-based Boston Area Community Health epidemiologic survey (5503 Boston residents, aged 30–79 years) revealing that the SES holds a more potent association with diabetes prevalence compared to ethnicity [95]. On the other hand, data from Australia have shown that all migrant groups exhibit higher T2DM prevalence compared to the Australian-born population across all SES strata [96]. As such, there is a growing need for further research into the sociodemographic factors related to high T2DM risk, which will take into account not only aging and ethnicity, but also the broader socioeconomic and socioecological determinants of prediabetes and T2DM [82, 83].

## Discussion

Currently, there are several clinical practice guidelines for T2DM screening and prevention which are endorsed by national and international organizations [97]. Accordingly, in clinical practice testing of adults for prediabetes and T2DM is typically performed based on predefined screening criteria related to established T2DM risk factors, such as those recommended by the American Diabetes Association (Table 2) [7]. Furthermore, numerous non-invasive risk scores (e.g. the Finnish diabetes risk score, FINDRISC), as well as extended models which additionally include biomarkers, have been developed in order to identify adults at high T2DM risk in the general population [10–12, 53]. Indeed, most non-invasive risk scores can identify individuals at high risk of developing T2DM within the subsequent 5- to 10-year period, with prediction models which include biomarkers performing slightly better [11]. However, most of these models may overestimate the actual T2DM risk [11], whilst data from the EPIC-InterAct study regarding non-invasive T2DM prediction models developed in populations of European ancestry showed that the performance of each model varies depending on age, sex, adiposity and country [98].

**Table 2:** Current criteria for testing for prediabetes or type 2 diabetes mellitus (T2DM) in asymptomatic adults, as recommended by the American Diabetes Association (ADA) [7]

ADA-recommended criteria for testing for prediabetes or T2DM in asymptomatic adults	
1. Testing should be considered in adults with body mass index (BMI) ≥25 kg/m^2^ (or ≥ 23 kg/m^2^ in Asian Americans) who have at least one of the following risk factors: (i) First-degree relative with diabetes (ii) Race/ethnicity related to high T2DM risk (iii) History of cardiovascular disease (iv) Hypertension (≥140/90 mmHg or on treatment for hypertension) (v) HDL-cholesterol plasma levels < 35 mg/dL (0.90 mmol/L) and/or triglyceride plasma levels > 250 mg/dL (2.82 mmol/L) (vi) Physical inactivity (vii) Women with polycystic ovary syndrome (PCOS) (viii) Other clinical conditions associated with insulin resistance (e.g. acanthosis nigricans)	
2. Annual testing should be performed in patients with prediabetes [i.e. impaired fasting glucose (IFG), impaired glucose tolerance (IGT), or HbA1C ≥5.7% (39 mmol/mol)]	
3. Lifelong testing at least every 3 years should be performed in women who were diagnosed with gestational diabetes mellitus (GDM)	
4. Testing should begin at the age of 45 years for all other individuals	
5. If results are normal, testing should be repeated at least every 3 years, with consideration for more frequent testing depending on the initial results and risk status.	

**Fig. 1:**
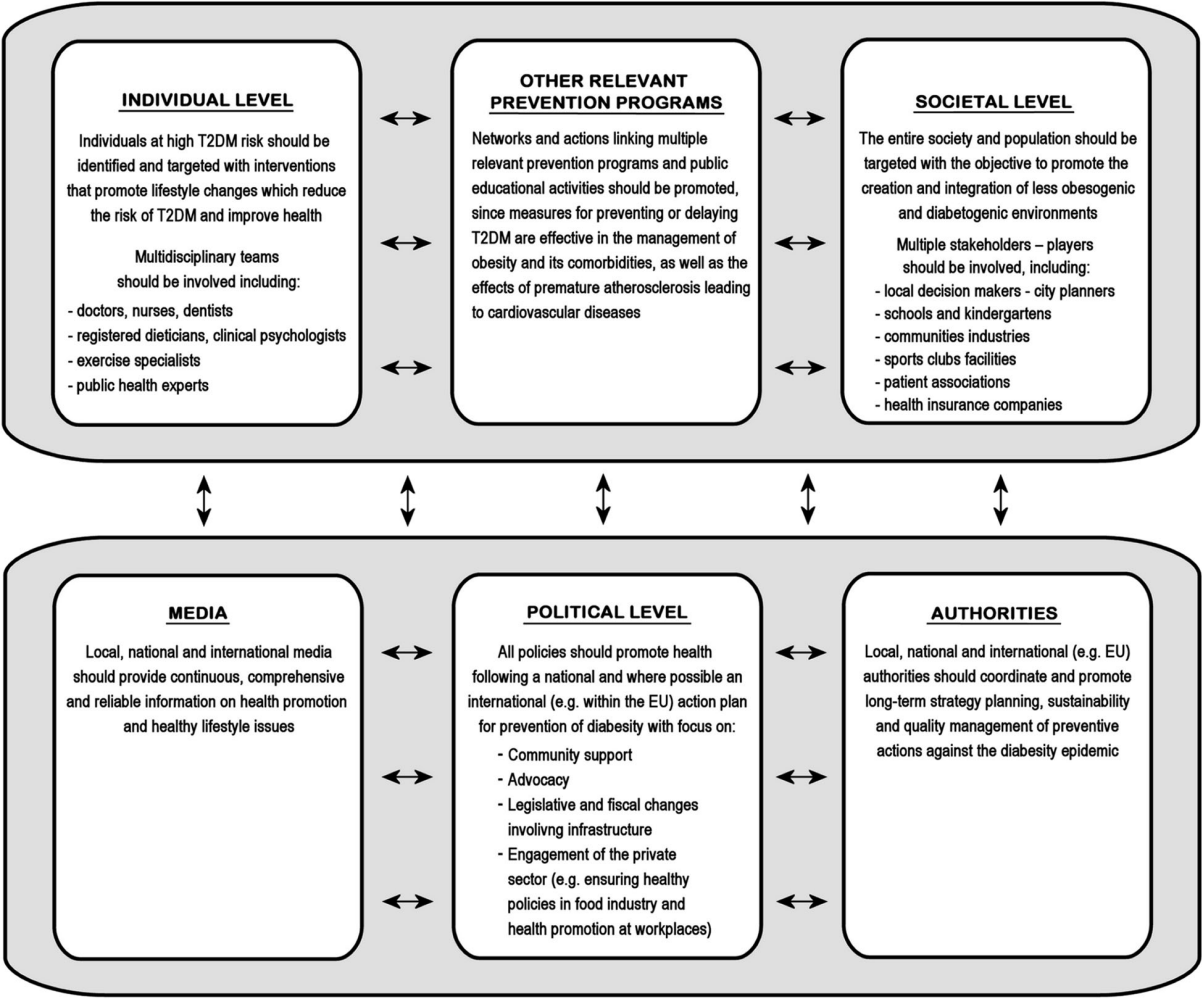
Prevention of type 2 diabetes mellitus (T2DM) in the context of a broader collaborative effort which mobilizes multiple partners/stakeholders at a national and international (e.g. European) level (adapted from Lindström et al. 2010 [53])

Moreover, it is important to highlight that, so far, the prevailing approach for T2DM prevention has been implementing a medical model for identifying and treating individuals at high T2DM risk through primarily one-by-one programs/interventions within the healthcare system [82, 83]. However, this model is considered to underestimate the complexity and scale of diabesity and fails to address vital underlying factors (e.g. low SES, deprivation and psychosocial stress) which increasingly fuel this problem [82, 83]. This is also clearly reflected in the disproportionate paucity of interventions aimed against fundamental social, economic and environmental factors which have been implicated in the current T2DM and obesity epidemic. Therefore, a broader strategic approach is required in order to confront the problem of T2DM prevention both at the individual and the societal level through a collaborative effort which will mobilize a wider range of stakeholders/partners (Fig. 1) [53].

Considering the current trends regarding aging, demographic structure, migration, and socioeconomic diversity across multinational and multicultural regions like Europe, this holistic approach is becoming increasingly essential in order to put into effect multidimensional public health programs and integrated interventions for effective T2DM prevention which will take into account both traditional and socioeconomic/socioecological factors. The EU-funded Feel4Diabetes study represents such an integrated approach, as it developed and implemented a school and community-based intervention which promoted healthy eating and active lifestyle for T2DM prevention in vulnerable families across six European countries (more than 12,000 families in Bulgaria, Hungary, Belgium, Finland, Greece and Spain) [13]. This was implemented through the provision of a supportive social and physical environment at multiple levels (i.e. home, school and municipality), as well as lifestyle counseling to the identified parents at high T2DM risk [13]. Of note, to make community-based approaches more cost-effective a two-stage screening process can be applied, initially targeting low SES groups and regions/municipalities with known higher prevalence of such groups (e.g. based on documented unemployment rates, income and education levels) which will be selectively screened with non-invasive scores (e.g. the FINDRISC). Subsequently, those individuals/families identified at high T2DM risk would be referred to local health centers for clinical screening/testing and interventions against modifiable T2DM risk factors. Overall, such multidimensional approaches for the prevention of T2DM may have a broader impact against the current diabesity epidemic within and across countries in Europe.

## Conclusions

In concluding this review, it is also important to acknowledge certain limitations of this work. As such, although we employed an effective bibliographic search strategy which aimed at reducing the bias in the selection of the reviewed articles, it should be acknowledged that not all relevant published papers have been included in this narrative review. However, the scope of this work was not to present an exhaustive list of all the relevant published studies or a systematic review on T2DM risk factors, but rather to provide a narrative overview of the relevant literature regarding key lifestyle-related and sociodemographic T2DM risk factors, particularly since the latter are often neglected. Furthermore, the emphasis of this specific work was placed on data from Europe which represents a region with high T2DM prevalence in combination with an increasingly aging population, multiple migrant/ethnic minority groups and significant socioeconomic diversity. Hence, although the aforementioned T2DM risk factors pose a global problem, it must be acknowledged that not all the findings/conclusions from the studies included in this review can be directly extrapolated and utilized to describe the exact association between each one of these risk factors and T2DM within the specific context of other regions of the globe. Finally, examining the contribution of the aforementioned risk factors to the complex pathophysiology of T2DM in children and adolescents was beyond the scope of the present review. Thus, readers with a specific interest in the growing problem of T2DM in children and adolescents, which appears to disproportionately impact ethnic/racial minorities, are encouraged to further consult additional reviews and consensus reports dedicated to this specific topic [99–101].

## Data Availability

Not applicable.

## Abbreviations

AHEI: Alternative Healthy Eating Index
BMI: Body Mass Index
DASH: Dietary Approaches to Stop Hypertension
DECODA: Diabetes Epidemiology Collaborative Analysis of Diagnostic Criteria in Asia
DECODE: Diabetes Epidemiology Collaborative Analysis of Diagnostic Criteria in Europe
EPIC: European Prospective Investigation into Cancer and Nutrition
FINDRISC: Finnish diabetes risk score
GDM: Gestational Diabetes Mellitus
GWAS: Genome-wide Association Studies
IDF: International Diabetes Federation
IFG: Impaired Fasting Glucose
IGT: Impaired Glucose Tolerance
PCOS: Polycystic Ovary Syndrome
RRR: Reduced Rank Regression
SES: Socioeconomic Status
T2DM: Type 2 Diabetes Mellitus
WHR: Waist to Hip Ratio
